# End-binding 1 protein overexpression correlates with glioblastoma progression and sensitizes to *Vinca*-alkaloids *in vitro* and *in vivo*

**DOI:** 10.18632/oncotarget.2646

**Published:** 2014-11-28

**Authors:** Raphael Berges, Nathalie Baeza-Kallee, Emeline Tabouret, Olivier Chinot, Marie Petit, Anna Kruczynski, Dominique Figarella-Branger, Stephane Honore, Diane Braguer

**Affiliations:** ^1^ Aix-Marseille Université, INSERM, CRO2 UMR_S 911, Marseille 13385, France; ^2^ APHM, CHU Timone, Marseille 13385, France; ^3^ Centre de Recherche d'Oncologie Expérimentale, Institut de Recherche Pierre Fabre, Toulouse, France

**Keywords:** glioblastoma, EB1, biomarker, *Vinca*-alkaloid, microtubules

## Abstract

End-binding 1 protein (EB1) is a key player in the regulation of microtubule (MT) dynamics. Here, we investigated the role of EB1 in glioblastoma (GBM) tumor progression and its potential predictive role for response to *Vinca*-alkaloid chemotherapy. Immunohistological analysis of the 109 human GBM cases revealed that EB1 overexpression correlated with poor outcome including progression-free survival and overall survival. Downregulation of EB1 by shRNA inhibited cell migration and proliferation *in vitro*. Conversely, EB1 overexpression promoted them and accelerated tumor growth in orthotopically-transplanted nude mice. Furthermore, EB1 was largely overexpressed in stem-like GBM6 that display *in vivo* a higher tumorigenicity with a more infiltrative pattern of migration than stem-like GBM9. GBM6 showed strong and EB1-dependent migratory potential. The predictive role of EB1 in the response of GBM cells to chemotherapy was investigated. Vinflunine and vincristine increased survival of EB1-overexpressing U87 bearing mice and were more effective to inhibit cell migration and proliferation in EB1-overexpressing clones than in controls. *Vinca* inhibited the increase of MT growth rate and growth length induced by EB1 overexpression. Altogether, our results show that EB1 expression level has a prognostic value in GBM, and that *Vinca*-alkaloid chemotherapy could improve the treatment of GBM patients with EB1-overexpressing tumor.

## INTRODUCTION

End-binding 1 protein (EB1) is an evolutionary conserved protein that preferentially localizes to the plus-ends of growing microtubules (MT). EB1 is the prototypic member of MT plus-end tracking proteins (+TIPs), which controls MT dynamics and links MTs to several cellular structures such as kinetochores and cell cortex [1–3]. EB1 directly interacts with many other +TIPs and is therefore central to the assembly of +TIPs complexes at MT plus-ends. With its binding partners, EB1 participates in MT-mediated cell functions, such as cell division, migration and morphogenesis. MT constitute a longstanding, important and effective target for anti-cancer drugs so-called Microtubule-Targeting Agents (MTA). MTA, including *Vinca*-alkaloids, taxanes and epothilones, are known to alter MT dynamic instability that is defined by growth to shrinkage transitions (catastrophes) and reverse transitions (rescues). However, the involvement of the proteins regulating MT plus-end dynamics in tumorigenesis and in drug response is still poorly understood. This question is very relevant in glioblastoma (GBM) cells, which motility is a microtubule-dependent and actin polymer-independent process [4]. We previously demonstrated that the anti-migratory effects of epothilone B on GBM cells occurred through an EB1-dependent mechanism and through MT catastrophe induction [5]. Such mechanism has also been described in GBM and endothelial cells with vinflunine (VFL) from the *Vinca*-alkaloid family [6]. A recent *in vitro* study with purified tubulin suggests that EB proteins sensitize MT to the action of MTA, by promoting MT catastrophes [7].

EB1 overexpression and its bad prognostic value have been described in several cancers, including breast cancer [8], esophageal squamous cell carcinoma [9], gastric adenocarcinoma [10], colorectal cancer [11] and hepatocellular carcinoma [12, 13].

GBM, the most common and malignant form of gliomas, is characterized by highly aggressive growth, and its invasive behavior that accounts for the poor overall survival (OS) of patients [14]. Current standard therapy following maximal safe removal consists of concomitant radio-chemotherapy with temozolomide (TMZ), an alkylating agent. Such regimen confers a median survival period of only 14.6 months and new therapeutic options are warranted [14]. *Vinca*-alkaloids are currently used in brain tumor treatment, more particularly vincristine (VCR), in combination with the alkylating agents procarbazine and lomustine for anaplastic oligodendrogliomas and oligoastrocytomas [15].

Here, we investigated the role of EB1 in GBM tumor progression and its potential predictive role for response to chemotherapy. We show that EB1 expression level has a prognostic value in GBM, and that *Vinca*-chemotherapy could improve the treatment of GBM patients with EB1-overexpressing tumor.

## RESULTS

### EB1 overexpression correlates with poor overall survival and progression-free survival in patients with GBM

**Figure 1:**
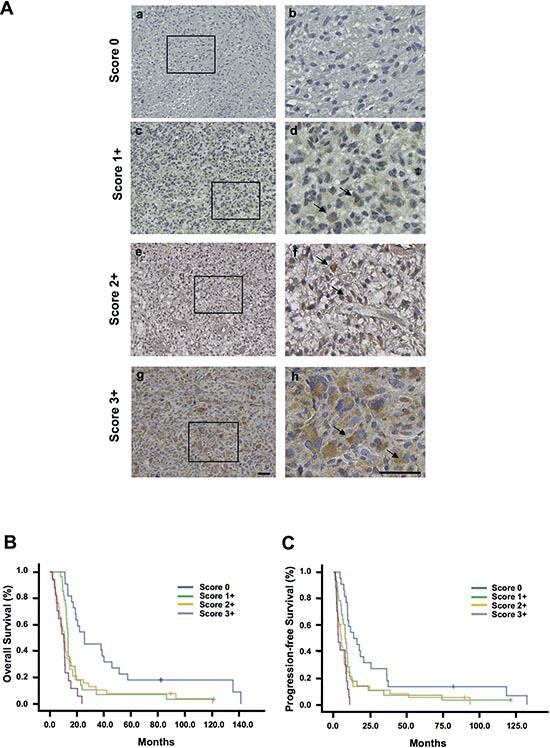
Prognostic relevance of EB1 expression in glioblastoma **(A)** Immunohistochemical staining of paraffin sections of glioblastoma tissue showing EB1 expression ranged from low to high: (a and b) score 0; (c and d) score 1+; (e and f) score 2+; and (g and h) score 3+. Arrows on magnified images show the cytoplasmic localization of EB1 in glioma cells (b, d, f, h). *Bar = 50 μm*. Kaplan–Meier overall survival **(B)** and progression-free survival **(C)** curves of 109 primary patients with different EB1 expression in glioblastoma tissue (score 0 to 3+).

EB1 expression was examined in human GBM tissue specimens coming from 109 GBM patient cohort (Table 1). Immunohistochemical analysis was performed using clone 5 anti-EB1 antibody (BD Bioscience) and isotype Ig as negative control. The EB1 expression appeared in the form of a cytoplasmic staining pattern (Fig. 1A). Scores were assigned as described in material and methods. EB1 staining and scores were validated by using another antibody against EB1 (clone H-70, Santa-Cruz Biotechnology) (not shown). Analysis of GBM tissue specimens revealed that out of the 109 tissues specimens examined, 22 were scored 0 (21%), 28 were scored 1+ (27%), 38 were scored 2+ (36%) and 17 were scored 3+ (16%). In univariate analysis, a higher EB1 expression was correlated to poor OS (*p* < 0.001) and poor PFS (*p* < 0.001) (Fig. 1B, C). Median OS and PFS for each EB1 scoring are shown in Supplementary table 1. By multivariate analysis adjusted by KPs and gender (Table 2), EB1 expression remained significant both for OS (*p* < 0.001, Hazard Ratio: 1.583) and PFS (*p* = 0.001, Hazard Ratio: 1.458).

**Table 1:** Main clinical characteristics of 109 GBM patients cohort

Characteristics	*N*	%
Median age, years (range)	62.1 (21.1–79.7)	
Male / Female	65/44	59.6/40.4
Median Karnofsky performance status (range)	70 (30–90)	
Extent of surgery		
- Complete resection	82	
- Partial resection	27	24.8
Radiotherapy	84	77.1
Median OS, months (range)	13.2 (1.5–141.4)	
Median PFS, months (range)	7.9 (0.9–132.4)	

**Table 2:** Univariate and multivariate analysis of PFS and OS

Univariate				Multivariate		
Factors	OS	PFS	OS	Hazard Ratio	PFS	Hazard Ratio
Age	0.172	0.402				
Extend of surgery	0.394	0.949				
KPS	0.05	0.238	0.028	1.65 (1.056–2.578)	0.217	
Gender	0.037	0.028	0.378		0.227	
EB1 expression	<0.001	<0.001	<0.001	1.583 (1.280–1.959)	0.001	1.458 (1.179–1.804)

KPS: Karnofsky Performans Status

When tested on lower grades of glioma, EB1 was not detectable or weakly expressed. Indeed, only 6.0% (2/33) pilocytic astrocytoma samples were positively stained (score 1+), and all anaplastic astrocytoma samples tested (0/40) were negative (score 0) (not shown).

Interestingly, the other proteins of EB1 family, EB2 and EB3, were expressed independently to EB1 scores in the 42 GBM tissue specimens analyzed (Supplementary Figure 1).

### EB1 expression correlates with GBM cell migration and proliferation

**Figure 2:**
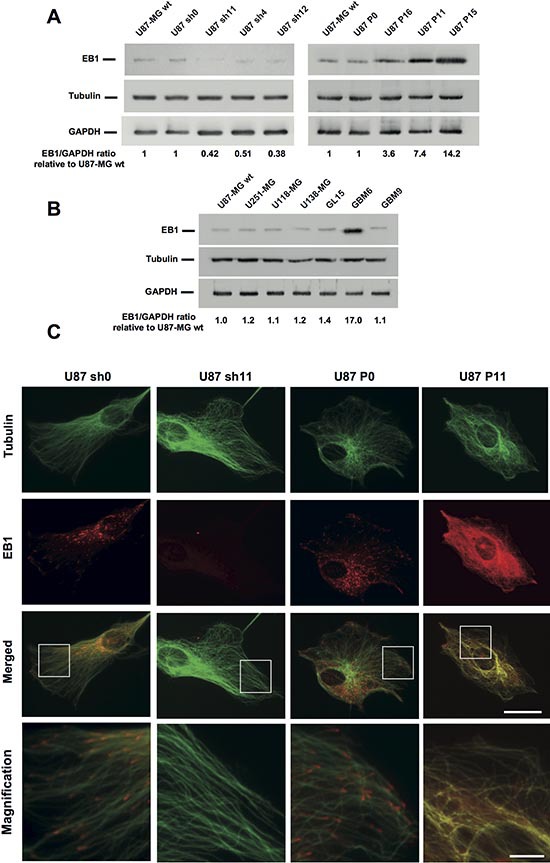
Modulation of EB1 expression in U87-MG cells Western blot analysis of EB1 levels in shRNA EB1 clones (U87 sh4, sh11 and sh12), EB1 overexpressing clones (U87 P11, P15 and P16) or control clones (U87-MG wt, sh0 and P0) **(A)**, and in GBM cell lines (U87-MG wt, U251-MG, U118-MG, U138-MG and GL15) or stem-like cells (GBM6 and GBM9) **(B)**. Ratios EB1/GAPDH, relative to U87-MG wt, from at least three independent experiments are presented under the blots. **(C)** Immunofluorescence staining of tubulin (green) and EB1 (red) in U87 sh0, sh11, P0 and P11 clones. *Bar = 10 μm*. Magnified images show modifications of EB1 distribution on MT. *Bar = 2 μm*.

In order to analyze the influence of EB1 expression in GBM tumor progression we generated six U87 stable clones deficient for EB1 (U87 sh4, sh11 and sh12) or overexpressing it (U87 P11, P15 and P16). Control clones U87 sh0 and P0 were generated with respective empty control vectors. EB1 expression level (ratio to GAPDH relative to U87-MG wt) in U87 sh4, sh11 and sh12 was around 2-fold lower than that in U87 sh0 control or U87-MG wt cells. Conversely, in overexpressing-EB1 clones U87 P16, P11 and P15, the EB1/GAPDH ratio relative to U87-MG wt was respectively 3.6, 7.4 and 14.2 fold higher than in U87 P0 control or U87-MG wt cells (Fig. 2A). Modulations of EB1 expression in clones had no effect on the protein level of other EB family members or on the level of tubulin (Supplementary Fig. 1). EB1 expression was confirmed in several GBM cell lines (U251-MG, U118-MG, U138-MG and GL15) and also in GBM stem-like cells isolated from 2 GBM patients (GBM6 and GBM9) [16] (Fig. 2B). Interestingly, EB1 expression level was almost 17-fold higher in GBM6 that is highly tumorigenic, as compared with GBM9 [17]. Finally, EB1 was not detected in human normal astrocytes (Supplementary Figure 2). Typical comet-like staining of EB1 was observed in U87 sh0 and P0 control cells (Fig. 2C). EB1 was barely visible in EB1 down-regulated clones as shown in U87 sh11, and stained in the entire length of MT in EB1 overexpressing clones without any MT bundling as illustrated with U87 P11.

**Figure 3:**
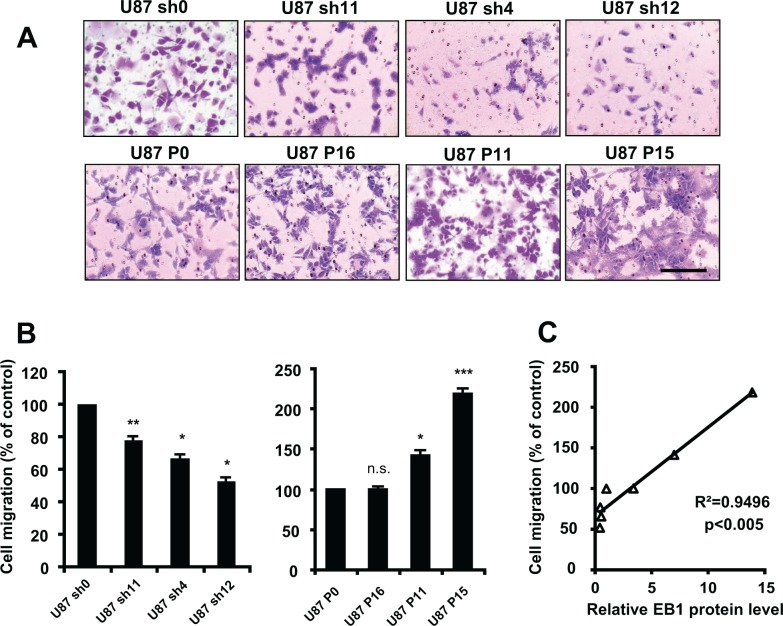

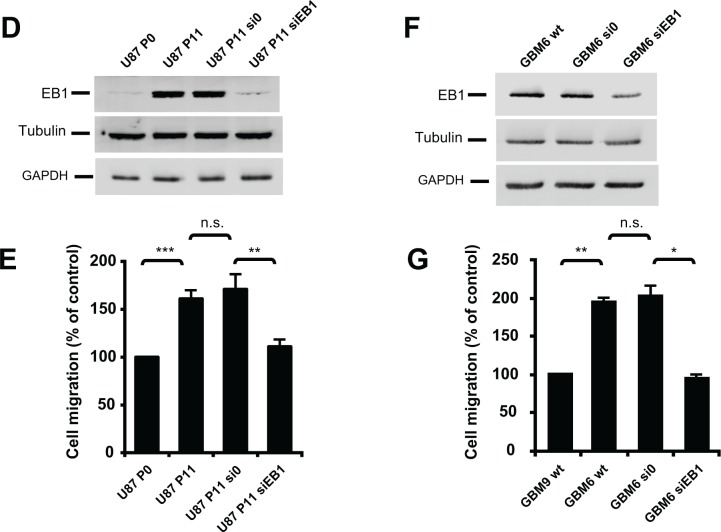
EB1 overexpression increases cell migration **(A)** Representative images of migratory EB1-down-regulating and -overexpressing U87-MG clones using the transwell migration assay (crystal violet staining, magnification of 100×). **(B)** Quantification of migratory cells from EB1-down-regulating and -overexpressing U87-MG clones, as determined by counting the cell number under the microscope with a magnification of 100×, in the transwell migration assay. **(C)** Correlation analysis between cell migration expressed as percentage of control clones and levels of EB1 expression in the different U87-MG clones expressed as relative ratios EB1/GAPDH expression. **(D)** Analysis of EB1 level expression by Western blot of U87 P11 cells treated with EB1 siRNA. U87 P11 cells were incubated or not (U87 P11) with EB1-specific siRNA (U87 P11 siEB1) and siRNA control (U87 P11 si0). **(E)** Quantification of migratory U87 P11 cells treated or not (U87 P11) with EB1-specific siRNA (U87 P11 siEB1) or siRNA control (U87 P11 si0), in the transwell migration assay. **(F)** EB1 level expression of GBM6 treated with EB1 siRNA. GBM6 were incubated or not (GBM6 wt) with EB1-specific siRNA (GBM6 siEB1) and siRNA control (GBM6 si0). **(G)** Quantification of migratory GBM6 treated or not (GBM6 wt) with EB1-specific siRNA (GBM6 siEB1) or siRNA control (GBM6 si0), in the transwell migration assay. At least three independent experiments were performed for each condition. Bar ± SEM. (*) indicates significant differences from control: *p < 0.05; **p < 0.005; ***p < 0.001, n.s.: non significant.

Implication of EB1 expression level in GBM cell migration was assessed by using a transwell assay (Fig. 3A, B). The knockdown of EB1 expression significantly decreased cell migration (−22.5 ± 2.8%, −33.2 ± 1.4% and −47.3 ± 2.4% for U87 sh11, sh4 and sh12, respectively), whereas overexpression of EB1 increased it (+41.6 ± 5.6% and +118.3 ± 6.2% for U87 P11 and P15, respectively). The level of EB1 expression in U87-MG clones was significantly correlated with their migrating potential (linear regression, R^2^ = 0.9496, *p* < 0.005) (Fig. 3C). Furthermore, introduction of siRNA against EB1 in EB1-overexpressing clones rescued the normal phenotype. Indeed, EB1 siRNA strongly reduced EB1 expression of U87 P11, 72 hours post transfection (Fig. 3D). EB1 silencing significantly decreased U87 P11 cell migration, which returned to U87 P0 basal level (percentage of migrating U87 P11 cells was reduced by 35.1 ± 4.4%) (Fig. 3E). Moreover, migration of astrocytes, with undetectable level of EB1, was significantly lower than U87-MG and U251-MG glioblastoma cells (−76.4 ± 4.5% versus U87-MG cells, *p* < 0.001) (Supplementary Figure 2C and D).

In addition, the involvement of EB1 in the migration of the two stem-like cells was investigated. We confirmed the strong migrating potential for GBM6 which was significantly higher than for GBM9 (194.5 ± 4.0% in GBM6 vs 100% for GBM9) (Fig. 3G) that has been previously described by using the wound-healing assay [16]. Moreover, EB1 down-regulation in GBM6 significantly abrogated migration by 52.9 ± 1.5% (Fig. 3F, G). Altogether, our data show that EB1 overexpression leads to a pro-migratory phenotype in U87-MG cells as well as in GBM stem-like cells.

**Figure 4:**
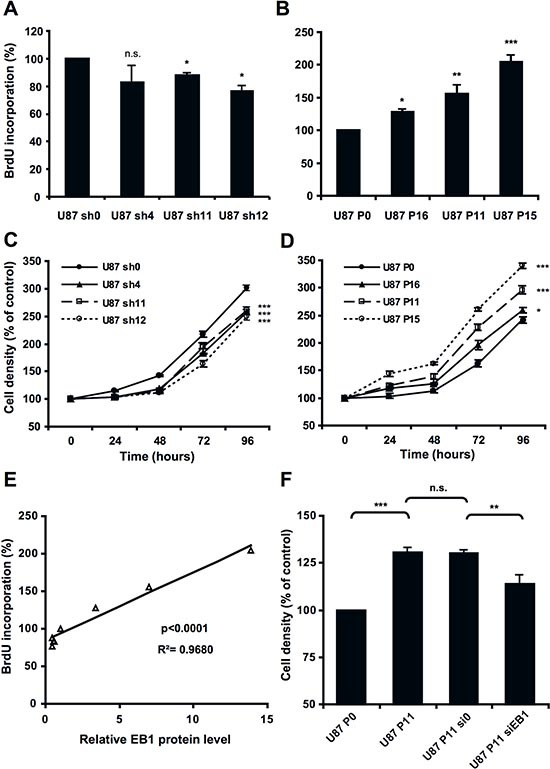
EB1 overexpression increases cell proliferation BrdU proliferation assay of down-regulating **(A)** or overexpressing **(B)** −EB1 clones in comparison with control U87 sh0 and P0 cells. Cell growth curves of down-regulating **(C)** or overexpressing **(D)** −EB1 clones in comparison with control U87 sh0 and P0 cells. **(E)** Correlation analysis between cell proliferation expressed as percentage of control clones and levels of EB1 expression in the different U87-MG clones expressed as relative ratios EB1/GAPDH expression. **(F)** Sulforhodamine B cell density assay at 72 hours of U87 P11 cells treated or not (U87 P11) with EB1-specific siRNA (U87 P11 siEB1) or siRNA control (U87 P11 si0). At each time, analyses were performed in sixplicates. At least three independent experiments were performed for each condition. Bar ± SEM. (*) indicates significant differences from control: *p < 0.05; **p < 0.005; ***p < 0.001, n.s.: non significant.

We next investigated the effect of EB1 expression on U87 cell proliferation using the BrdU staining assay. As shown in Fig. 4A, shRNA-mediated knockdown of EB1 expression slightly inhibited cell proliferation (−12.3 ± 0.9%, *p* < 0.05 and −23.5 ± 3.7%, *p* < 0.05 for U87 sh11 and sh12, respectively). Conversely, EB1 overexpression significantly promoted cell proliferation (+28.3 ± 2.1%, +55.7 ± 11.3% and +104.1 ± 8.2% for U87 P16, P11 and P15, respectively) (Fig. 4B). Together, these data revealed a positive and statistically significant correlation between the level of expression of EB1 in U87-MG cells and their proliferation rate (linear regression, R^2^ = 0.9680, *p* < 0.001) (Fig. 4E), suggesting that EB1 is involved in U87 cell proliferation. The EB1 expression-dependent effect on proliferation was confirmed on cell growth by using the sulforhodamine B staining assay (Fig. 4C and D). Indeed, in the 3 shRNA clones with 2-fold lower EB1 expression, the doubling time was increased by around 12 % and cell number was significantly decreased by around 15% at 96 hours. In contrast, EB1 overexpression decreased the doubling time in a EB1-dependent manner (−1%, −16.6% and −25.9% for U87 P16, P11 and P15, respectively) and significantly promoted cell growth (+6.4 ± 2.5%, +21.6 ± 3.0% and +39.6 ± 1.7% for U87 P16, P11 and P15, respectively) (Fig. 4D). These data were confirmed using a trypan blue dye exclusion assay (data not shown). Moreover, down-regulation of EB1 significantly decreased viability of U87 P11 cells by 12.5 ± 3.3%, thus rescuing partially the normal U87 P0 phenotype (Fig. 4F). Proliferation of astrocytes, that display undetectable staining of EB1, was also significantly lower than U87-MG and U251-MG glioblastoma cells (−59.61 ± 8.6% versus U87-MG cells at 96 hours, *p* < 0.001) (Supplementary Figure 2). Our results show that besides cell migration, EB1 expression level affects GBM cell proliferation.

### EB1 overexpression increases tumor growth in an orthotopic mouse model of GBM

**Figure 5:**
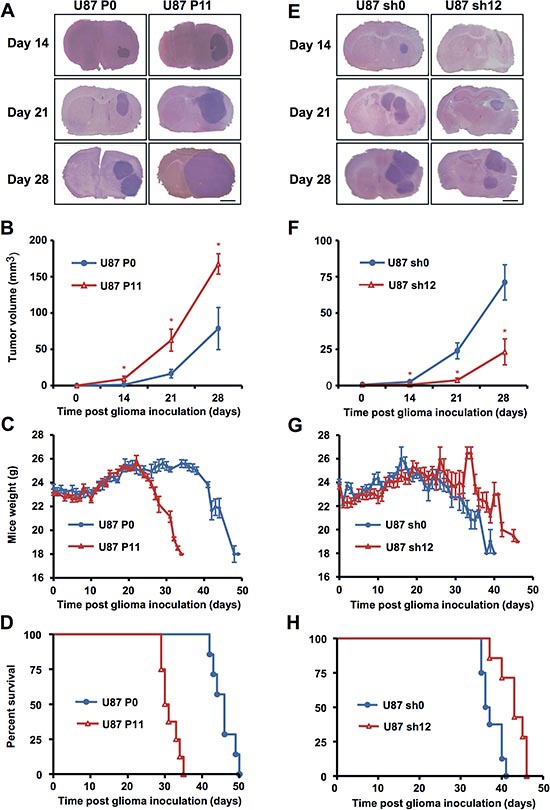
EB1 expression modulates tumor growth in orthotopic nude mouse model Typical coronal sections of brains from mice intracerebrally grafted with EB1-overexpressing U87 P11 cells **(A)** or EB1 down-regulated U87 sh12 cells **(E)** in comparison with control U87 P0 (A) and U87 sh0 cells (E). Brains were recovered at indicated days following cell transplantation. *Bar = 1 mm*. **(B, F)** Tumor volumes (mean ± SEM of 3 animals per treatment group). *p < 0.05. **(C, D)** Mean weights (Bar ± SEM). **(D, H)** Kaplan–Meier survival plot of tumor-grafted mice.

To confirm *in vivo* the effect of EB1 on GBM tumor progression, control U87 P0 or EB1 overexpressing U87 P11 clones were transplanted by stereotaxy in the striatum of nude mice. At post transplant days 14, 21 or 28, animals were euthanized and serial coronal brain sections were stained with HE, then the size of the tumor was calculated by morphometry by using all the sections. Relative to control tumors, those overexpressing EB1 were significantly more extended and the volume was increased at post transplant day 14 by 682.2 ± 316.1%, day 21 by 281.7 ± 32.9% and day 28 by 113.3 ± 17.2% (Fig. 5A, B). Ki-67 staining confirm that EB1 overexpression increased proliferation rate of U87 P11 tumors (+56.5 ± 8.2 % versus U87 P0 tumors, *p* < 0.05) (Supplementary Figure 3). The clinical condition of the mice was monitored daily. Weight loss was more rapid for mice bearing overexpressing EB1 tumors (Fig. 5C). Consequently, survival for these animals was strongly and significantly reduced (30.5 days for U87 P11 bearing mice *vs* 46 days for controls, *p* < 0.001) (Fig. 5D). Our previous work showing that GBM6 which overexpress EB1 gave rise to huge tumors when injected into brain, strengthens the current study [17].

Conversely, when mice were grafted with EB1 down-regulated U87 sh12 clone, size of tumors was significantly reduced at post transplant days 14, 21 and 28 (−98.0 ± 1.4% at day 14, −86.8 ± 6.3% at day 21 and −62.3 ± 14.9% at day 28, as compared with control U87 sh0 bearing mice) (Fig. 5E, F). Weight loss was delayed and survival was significantly higher (survival gain of 6.5 days, *p* < 0.005) (Fig. 5G, H).

### Response to Vinca-alkaloid treatment is enhanced in animals with EB1 overexpressing GBM cells orthotopically xenografted

To investigate *in vivo* the influence of EB1 overexpression on drug response, VCR, a MTA used in the treatment of gliomas, and VFL, a new member of the *Vinca*-alkaloid family were tested. Control U87 P0 or EB1 overexpressing U87 P11 cells were transplanted by stereotaxy to the striatum of nude mice and animals were treated or not with VCR or VFL.

**Figure 6:**
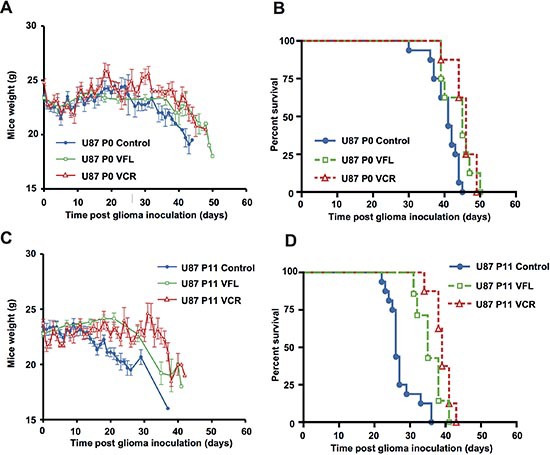
EB1 overexpression sensitizes glioblastoma cells to *Vinca*-alkaloid anti-tumor growth effect Mean weights **(A, C)** and Kaplan–Meier survival plot **(B, D)** of control U87 P0 or EB1-overexpressing U87 P11 bearing mice intravenously treated with VFL, VCR or vehicle. Bar ± SEM.

A significant survival benefit confirmed by mice weight analysis was observed when EB1 overexpressing U87 P11 bearing mice were treated with a *Vinca*-alkaloid, whatever the drug (Figure 6). The higher survival gain (13 days, *p* < 0.001) was obtained with VCR. Indeed, median survival was 39 days and 26 days for U87 P11 bearing mice treated with VCR and vehicle, respectively (Fig. 6D). Survival gain was only 5 days in control U87 P0 bearing mice treated in the same conditions (median survival of 46 and 41 days after VCR and vehicle treatment, respectively) (Fig. 6B). Tumor volume measurements confirm these data. VCR strongly reduced tumor volumes in U87 P11 bearing mice (−90.5 ± 3.0% vs vehicle, *p* < 0.005) as compared with U87 P0 bearing mice (−62.2 ± 11.2% vs vehicle, *p* < 0.05) (Supplementary Figure 4). Proliferation rate was significantly reduced in U87 P11 cells but not in U87 P0 cells after VCR treatment, as shown by Ki-67 staining (−46.2 ± 7.0% vs vehicle, *p* < 0.005, at day 21) (Supplementary Figure 3).

A significant survival benefit (9 days, *p* < 0.005) was also obtained with VFL. Indeed, median survival of EB1 overexpressing U87 P11 bearing mice was 35 days and 26 days, after VFL and vehicle treatment, respectively (Fig. 6D). In contrast, survival of control U87 P0 bearing mice was not improved after VFL treatment (median survival of 45 and 41 days, after VFL and vehicle treatment, respectively) (Fig. 6B).

In contrast to VCR and VFL, which display an enhanced survival gain (+55 and 60% respectively) in EB1 overexpressing U87 P11 bearing mice, temozolomide (TMZ), the reference drug in GBM, induced a better survival gain in low EB1expressing U87 P0 bearing mice (48 days and 24 days, in U87 P0 and U87P11, respectively (Supplementary Figure 5). Taken together, our results show that EB1 expression, despite its bad prognostic value level, may be considered as a predictive marker for *Vinca*-alkaloid response in mice models.

### EB1 overexpression sensitizes GBM cells to Vinca-alkaloid antimigratory and cytotoxic effects

**Figure 7:**
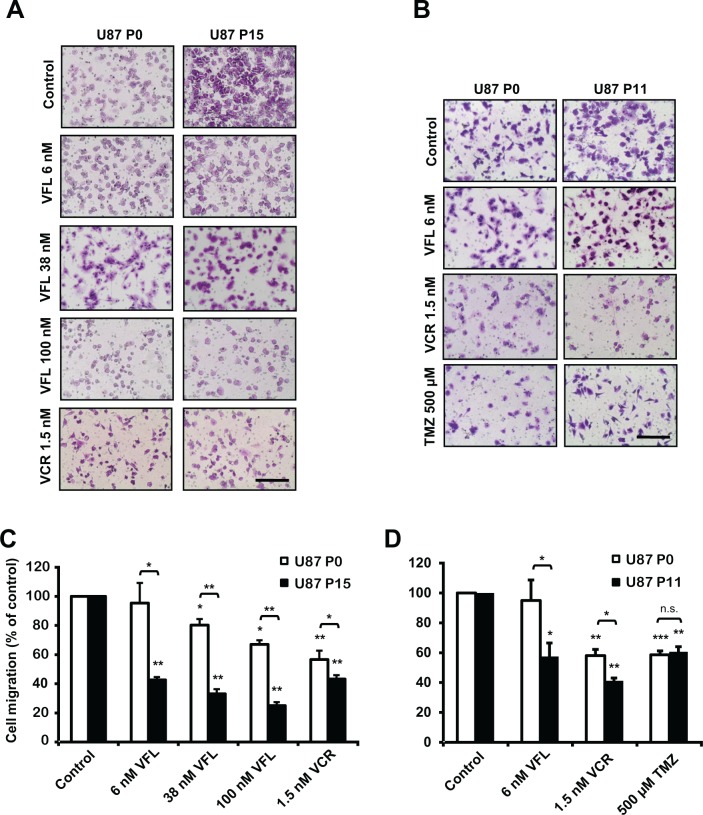

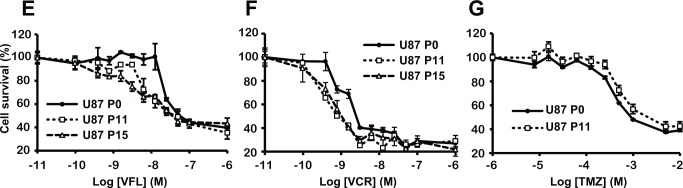
EB1 overexpression sensitizes glioblastoma cells to *Vinca*-alkaloid anti-migratory and cytotoxic effects **(A)** Representative images of U87 P0 and U87 P15 migratory cells treated or not (control) with VFL (6, 38 and 100 nM) or 1.5 nM VCR using the transwell migration assay (crystal violet staining, magnification of 100×). **(B)** Representative images of U87 P0 and U87 P11 migratory cells treated or not (control) with 6 nM VFL, 1.5 nM VCR or 500 μM TMZ using the transwell migration assay (crystal violet staining, magnification of 100×). **(C)** Quantification of migratory cells treated or not (control) with VFL (6, 38 and 100 nM) or 1.5 nM VCR, in the transwell migration assay. **(D)** Quantification of migratory cells treated or not (control) with 6 nM VFL, 1.5 nM VCR or 500 μM TMZ, in the transwell migration assay. Quantification was expressed as percentage of migrating cells relative to 100% of control cells. At least three independent experiments were performed for each condition. Bar ± SEM. (*) indicates significant differences from control: *p < 0.05; **p < 0.005; ***p < 0.001, n.s.: non significant. Dose response curves of the cytotoxicity of VFL **(E)**, VCR **(F)**, or TMZ **(G)** in overexpressing-EB1 U87 P11 and/or U87 P15 clones, in comparison with control U87 P0 cells. At least three independent experiments were performed.

For deciphering how EB1 overexpression influences GBM cell response to *Vinca*-alkaloid treatment, we assessed the effect of EB1 expression on the anti-migratory properties of both VFL and VCR. VFL inhibited U87 P0 and U87 P15 cell migration in a dose-dependent manner (Fig. 7A, C). Interestingly, inhibition was more pronounced in EB1 overexpressing U87 P15 clone. Indeed, 6 nM VFL failed to alter control cell migration whereas it inhibited U87 P15 cell migration (−57 ± 1.9%). This result has been confirmed with EB1 overexpressing U87 P11 clone (−39.4 ± 9.5%) (Fig. 7B, D). Moreover, at higher concentrations of VFL, inhibition of migration was more efficient in U87 P15 than in U87 P0 cells (Fig. 7A, C). These data demonstrate that EB1 expression level in GBM cells modulates VFL anti-migratory effect. EB1 overexpression also sensitized cells to VCR in U87 P15 cells (−56.7 ± 2.1% vs −43.3 ± 6.0% for U87 P15 and U87 P0, respectively) (Fig. 7A, C) and in U87 P11 cells (−60.0 ± 1.9 % vs −41.9 ± 4.1 % for U87 P11 and U87 P0, respectively) (Fig. 7B, D). In contrast, we did not detect any significant difference of the anti-migratory effect of TMZ according to EB1 expression (Fig. 7B, D).

Further, we conducted a dose response cytotoxic activity assay of VFL on the two overexpressing-EB1 U87-MG clones after 72 hours of treatment (Fig. 7E). The drug concentration required to reduce viability by 50% (EC_50_) was 10.6 ± 1.8 nM for U87 P11 and 7.0 ± 1.8 nM for U87 P15 in comparison with 38.0 ± 1.5 nM for control U87 P0. Moreover, the concentration of VCR required to reduce viability by 50% (EC_50_) was 0.60 ± 0.22 nM for U87 P11 and 0.61 ± 0.10 nM for U87 P15 in comparison with 1.42 ± 0.05 nM for control U87 P0 (Fig. 7F). Conversely, we did not detect any difference of cytotoxicity after TMZ treatment in the same experimental conditions (EC_50_ = 500.3 ± 162.4 μM and 578.6 ± 152.1 μM for U87 P0 and U87 P11 cells respectively) (Fig. 7G). These data obtained *in vitro* supports the results from the *in vivo* study, and demonstrate that EB1 overexpression specifically sensitizes to *Vinca*-alkaloids anti-migratory and cytotoxic effects in GBM cells.

### Vinca-alkaloids antagonize the alteration of MT dynamics induced by EB1 overexpression

In order to decipher the cellular mechanism of *Vinca*-alkaloid sensitization, we analyzed the effects of VCR on MT dynamic instability from EB3-GFP comet data in control U87-MG cells and U87 EB1 overexpressing cells (Table 3). First, our results clearly show that EB1 overexpression increased the MT growth rate (+59.6%, *p* < 0.01), and strongly decreased the distance-based catastrophe frequency (−41.3 %, *p* < 0.01). Analysis of microtubule dynamics from CLIP-170-GFP comet data confirmed the decrease in the distance-based catastrophe frequency in U87 P15 versus U87 P0 cells (−20%, *p* < 0,05). In U87 P0 cells, VCR did not alter the growth rate and catastrophe frequency, excepted at the higher concentration (1.6 nM). Indeed, this cytotoxic and anti-migratory concentration slightly increased the MT catastrophe frequency (+23.2%, *p* < 0.05, for 1.6 nM). In contrast, in U87 P11, VCR strongly increased the distance based-catastrophe frequency at all the concentrations (+52.3%, *p* < 0.05 at 0.8 nM, + 64.4%, *p* <0.01 at 1.2 nM and +69.2%, *p* < 0.01, at 1.6 nM) and statistically decreased the MT growth rate (−31.1 %, *p* < 0.05 at 1.2 nM and −27.9%, *p* < 0.05 at 1.6 nM). These results suggest that VCR antagonize the alterations of MT dynamics induced by EB1 overexpression. Samples videos are presented in Supplemental movies 1 to 4.

**Table 3:** Microtubule dynamic instability parameters from EB3-GFP or CLIP-170-GFP comet tracks

	Growth Rate (μm/min)	Growth Length (μm)	Growth Time (min)	Catastrophe Frequency (μm^−1^)	Catastrophe Frequency (min^−1^)
**U87 P0-EB3-GFP Control**	7.45 ± 0.75	1.82 ± 0.16	0.25 ± 0.01	0.55 ± 0.05	3.93 ± 0.11
0.8 nM VCR	7.82 ± 0.51	1.86 ± 0.13	0.24 ± 0.005	0.54 ± 0.04	4.09 ± 0.08
1.2 nM VCR	7.36 ± 0.13	1.66 ± 0.02	0.23 ± 0.006	0.6 ± 0.01	4.26 ± 0.11
1.6 nM VCR	6.53 ± 0.25	1.48 ± 0.06	0.24 ± 0.004	0.68 ± 0.03*	4.23 ± 0.06
**U87 P11-EB3 –GFP Control**	11.89 ± 1.31^##^	3.11 ± 0.34^##^	0.26 ± 0.01	0.32 ± 0.04^##^	3.81 ± 0.16
0.8 nM VCR	10.24 ± 0.61	2.04 ± 0.20*	0.21 ± 0.014*	0.49 ± 0.05*	4.84 ± 0.32*
1.2 nM VCR	8.19 ± 0.67*	1.89 ± 0.11**	0.24 ± 0.013*	0.53 ± 0.03**	4.11 ± 0.22*
1.6 nM VCR	8.58 ± 0.23*	1.84 ± 0.03**	0.23 ± 0.002*	0.54 ± 0.01**	4.41 ± 0.04*
**U87 P0-CLIP170-GFP**	11.0 3± 0.92	2.41 ± 0.12	0.22 ± 0.01	0.41 ± 0.02	4.55 ± 0.27
**U87 P15-CLIP170-GFP**	12.32 ± 1.02	3.03 ± 0.23^#^	0.24 ± 0.01	0.33 ± 0.03^#^	4.17 ± 0.15

# *p* < 0.05;

## *p* < 0.01 (vs P0);

* *p* < 0.05;

** *p* < 0.01 (vs Control)

## DISCUSSION

In this study, we identified EB1 as a potential prognostic marker in GBM patients and a predictive factor of response to *Vinca*-alkaloid treatment. Moreover, the prognostic impact on OS and PFS was shown to be independent of other known prognostic factors including age, functional status, and surgery. Further studies for testing EB1 in addition to other biomarkers such as MGMT status and IDH1 mutation are warranted in patients with GBM and other high grade gliomas to confirm EB1 prognostic role [18–22].

By modulating EB1 expression in U87-MG cells, we have shown that EB1 promotes GBM cell proliferation and migration, thus confirming the well-described role of EB1 in the mechanism of cell migration [23–25] and cell division [8, 9, 26, 27]. Furthermore, we show for the first time that EB1 promotes GBM tumor growth in nude mice. Analysis of the GBM-derived stem-like cells GBM6 and GBM9 strengthen these observations. GBM6 that strongly overexpressed EB1 in contrast to GBM9, showed an EB1-dependent and strong migratory potential, and a higher tumorigenicity with a more infiltrative pattern *in vivo* [17]. In addition, gene expression profiles evidenced a distinctive gene expression signature for GBM6 and GBM9, characterized by a specific overexpression of genes involved in cell migration and adhesion for GBM6. However, when this analysis was performed, EB proteins were not selected for migration signature. Based on their original brain location and their distinctive molecular signature, GBM6 and GBM9 have been classified as mesenchymal and proneural GBM subtypes respectively [17]. Since EB1 overexpression seems to be specific to GBM6, EB1 expression profile should be included to the other known distinctive genes characterizing mesenchymal GBM, and may help to define new subtypes.

Interestingly, our study allows us to add EB1 among the proteins regulating the MT cytoskeleton that are overexpressed in GBM such as spastin, class III β-tubulin and γ-tubulin [28, 29]. Further work is needed to decipher the interactions between these proteins. For example, γ-tubulin complexes and EB1 have been shown to produce apparent antagonistic effect on MT dynamics and spindle positioning in HeLa Cells [30]. However, such interaction may be interpreted only after a deep knowledge of EB1 effect on MT dynamic instability, taking into account several factors such as EB1 expression level and post translational modifications like phosphorylation and detyrosination [31–33].

Recent publications indicate that EB1 may play a role in tumorigenesis suggesting that beyond its prognostic value, EB1 could be considered as a potential oncogene. EB1 induces cell proliferation through the activation of beta-catenin/Tcell factor pathway [9, 21], and the activation of aurora-B [34], a kinase essential for mitosis in breast cancer for which EB1 negatively impacts survival [8]. Interestingly, aurora-B expression correlates with GBM aggressive behavior [27]. It has also been shown that abnormal detyrosination of the tubulin C-terminal EEY sequence promotes tumor cell growth, which represents a significant marker of tumor aggressiveness in breast cancer [35]. We recently found that EB1, which shares the same C-terminal sequence as tubulin [36, 37], undergoes a C-terminal detyrosination process in GBM cells, which also may account for enhanced tumor progression [6]. Furthermore, tubulin detyrosination as well as EB1 detyrosination, are known to impair accumulation of CAP-Gly proteins at growing MT plus-end, thus altering MT dynamics and related cell functions [37, 38].

Finally we demonstrated that EB1 overexpression sensitizes GBM cells to *Vinca*-alkaloids by enhancing anti-migratory and cytotoxic effects. However, EB1 overexpression in GBM cells did not improve the response to TMZ. These results suggest that the mechanism of sensitization by EB1 is MT-dependent. Indeed, we demonstrated that *Vinca*-alkaloids alter so potently MT dynamics in EB1 overexpression that they completely reverse the phenotype due to EB1 overexpression. Our results are consistent with a recent publication describing *in vitro* a mechanism of cooperation between EBs and MTA [7]. Indeed, in the presence of EB proteins, MT growth rates were inhibited and MT catastrophes were increased at concentrations of drugs that did not affect MT dynamics in the absence of EB. The authors suggested that catastrophes were MT age-dependent, and that MT depolymerizing drugs accelerate aging in an EB-dependent manner. Moreover, we previously reported in U87-MG cells that anti-migratory effect of VFL was associated with the inhibition of EB1 accumulation at MT plus-end and with the decrease of C-ter detyrosinated form of EB1 [6]. This shift in post-translational modification of EB1 may participate to *Vinca*-alkaloid antitumor activity, if one considers that detyrosinated EB1 promotes tumor progression as described with detyrosinated tubulin [35]. It is also conceivable that increased cell proliferation may sensitize the cells to the action of cell cycle-dependent anticancer drugs.

Altogether, our data open new avenues of research on the role of EB1 in GBM tumor progression that has limited therapeutic options. Subgroups of glioma patients with EB1 overexpressing tumor should be identified. Furthermore, MT targeting drug activity should be evaluated in regard to EB1 status in cancer patients.

## MATERIALS AND METHODS

### Drugs

Vinflunine (Pierre Fabre France) and vincristine (Sigma-Aldrich) were solubilized in sterile distilled water or in a saline solution. Temozolomide was extracted from 5 mg Temodal® capsules in dimethyl sulfoxide (DMSO).

### Patients and tissue samples

We analyzed 109 patients with GBM referred to our institution. Seventy-two of them were included in tissue microarrays (TMA) that were constructed from formalin-embedded tumor material. Brain tumor samples were classified according to WHO CNS tumor classification [39] and their main clinical features are reported in Table 1. Areas of viable and representative tumor following review of all blocks were marked by two pathologists prior to inclusion into the TMA (3 × 0.6 mm cores for each tumor) [40, 41]. Consensual prognostic factor such as age, functional status analyzed by Karnovsky Performance Score (KPS), and surgery were collected. Patients were routinely follow-up with brain MRI every two months and assessment of progression was performed according to MacDonald criteria's [42]. TMA containing tissue from 33 patients with pilocytic astrocytoma (WHO grade 1) and 40 patients with anaplasic astrocytoma (WHO grade 3) were also analyzed. The study was undertaken after informed consent from each patient or their relatives, in accordance with institutional board guidelines.

EB1 immunohistochemistry was carried out, using an anti-EB1 primary antibody (clone 5, BD Biosciences) [8, 9], and avidin–biotin–peroxidase method [43]. Isotype control antibody (BD Biosciences) was used at the same concentration as primary antibody. For confirmation, a second anti-EB1 primary antibody was also used (clone H-70, Santa-Cruz Biotechnology) [13]. EB2 and EB3 immunohistochemistry was performed using an anti-EB2 primary antibody (Abcam, ab56753), and an anti-EB3 primary antibody (Abcam, ab157217). Positive cells were scored based on cytoplasm staining of EB1. Only tumor cell staining was taken into account, excluding hypertrophic endothelial cells in tumor blood vessels. The number of positive immunostained cells out of the total number of tumor cells (> 1,000) in 20 non-overlapping high power fields (30,000 μm^2^, objective 40X) per clinical sample was analyzed. EB1 expression was classified semi-quantitatively according to the visual scoring as 0 (undetectable staining in tumor cells), 1+ (<10% of tumor cell staining), 2+ (10%–50% of tumor cell staining, or 3+ (>50% of tumor cell staining). Manual cell counting of labeled tumor cells was performed by 2 pathologists independently, without knowledge of the patient clinical status. The coefficient of variation of interobserver reproductibility was 9.68% for EB1 expression scoring, with no significant difference between the two observers (paired t-test, *p* = 0.88).

### Cell culture and transfection

Human glioblastoma U87-MG cell line was ordered from ATCC (number HTB14). Human glioblastoma U118-MG, U251-MG, U138-MG and GL15 cells were kindly provided by D. Figarella-Branger (Pathology and Neuropathology Unit and Tumour Bank). U87-MG, U251-MG, U118-MG, U138-MG and GL15 cells were grown in EMEM media with glucose and L-glutamine (Lonza, Levallois-Perret, France), containing 10% fetal calf serum (Lonza), 1% penicillin/streptomycin (Sigma-Aldrich, Saint-Quentin Fallavier, France). GBM stem-like cell lines GBM6 and GBM9 were isolated from GBM patients and grown as previously described [16]. Normal human astrocytes were purchased from Lonza and cultured in Astrocytes Basal Media supplemented with astrocyte growth medium SingleQuots (Lonza). All cell types were tested weekly for the presence of mycoplasma, using Mycoalert^TM^ Mycoplasma Detection Kit (Lonza).

ShRNA plasmid that specifically knocked out human EB1 (NM_012325) and negative shRNA control plasmid (Mission® non-target shRNA control vector) were obtained from Sigma-Aldrich. EB1 expression plasmid (16474) and negative control plasmid (16440) were obtained from Addgene. U87-MG cells were transfected with lipofectamineTM 2000 system (Invitrogen). For establishing stable clones, the shRNA-transfected cells and related control clones were selected in culture medium containing puromycin (Sigma-Aldrich) 24 hour post-transfection. EB1-overexpressing clones and related control clones were selected in 800 μg/ml G418. For EB1 silencing by transient transfection, cells were transfected by lipofectamine 2000 system with siRNA for EB1 (Hs_MAPRE1-5, Qiagen) and all STAR control (Qiagen). EB1 down-regulation was analyzed 48 hours or 72 hours later by western blotting.

### Immunofluorescence and western blot analysis

Indirect immunofluorescence was performed as previously described [5] by using the anti-EB1 antibody and anti-mouse antibody Alexa 568 nm (Molecular Probes); and FITC-coupled anti-α-tubulin antibody (clone DM1A; Sigma-Aldrich). Cells were observed using a Leica DM-IRBE microscope, 100X magnification. All images were acquired using Metamoph software (Molecular Devices, Sunnyvale, CA) at identical acquisition settings, and were processed using Image J software. After cell lysis, 30 μg of total protein were loaded into a 12% SDS-PAGE gel. Anti-EB1 antibody, anti-α-tubulin and anti-mouse IgG-horseradish peroxidase (Jackson Immunoresearch) were used. Chemiluminescence detection kit (Millipore) was used for visualization of protein bands. Chemiluminescent signal was acquired on a G:BOX imaging system (Syngene, Cambridge, UK) and quantification was done with Image J software.

### Analysis of cell proliferation

Cell proliferation was assessed using 5-bromodeoxyuridine (BrdU) immunohistochemistry as previously described [44]. After 4-hour incubation with 3 μg/mL BrdU (Sigma-Aldrich), cells were labelled with monoclonal anti-BrdU antibody (Sigma-Aldrich) followed by Alexa 568 nm anti-mouse antibody (Molecular Probes). Nuclei were stained with 4′6-diaminido-2-phenylindole (DAPI; Sigma-Aldrich). The stained cells were observed under microscope, 20X magnification. Minimums of 200 cells were scored for BrdU incorporation. Cell growth analysis was performed using the sulforhodamine B assay as previously described [45]. For cytotoxicity assay, drugs were added to the medium 24 h after cell seeding. At least three independent experiments were performed for each condition.

### Transwell migration assay

Cells (5 × 10^4^ cells/well) were seeded on the upper side of transwell migration chamber (Becton Dickinson, Le Pont de Claix, France) and allow migrating for 5 hours as previously described [5]. Six fields per condition were imaged and transmigrated cells were counted. Results were expressed as percent of transmigrated cells compared with no treatment condition. At least three independent experiments were performed for each condition.

### Animal studies

#### Intracerebral tumor transplantation

All experimental procedures and animal care were carried out in conformity with the guidelines of the French Government and approved by the Regional Committee for Ethics on Animal Experiments. Six to 8 weeks old female Swiss nude mice were obtained from Charles River Laboratories France (L'Arbresle, France). After animal anesthesia, 5 × 10^5^ tumor cells were injected into the striatum of the mice, as previously described [46].

#### Experimental design

To evaluate consequences of the level of EB1 expression in GBM progression, 4 groups were constituted: mice bearing U87 P0 (*n* = 17), U87 P11 (*n* = 17), U87 sh0 (*n* = 17) and U87 sh12 (*n* = 17). For analysis of EB1-overexpression on antitumor effects of drugs, groups of U87 P0 and U87 P11 bearing mice were constituted, and received VCR (*n* = 14), VFL (*n* = 14), TMZ (*n* = 14) or vehicle solutions (control groups) (*n* = 14).

#### Treatments

VFL (20 mg/kg/day) or VCR (0.8 mg/kg/day) treatments were administered intravenously at days 4, 7, 11, 14, 18 and 21 after glioma implantation according to the treatment schedule previously described [47] and preliminary tolerance studies (not shown). VFL and VCR were dissolved at 2.5 mg/ml and 0.16 mg/ml respectively in 0.9% NaCl. TMZ (5 mg/kg) was administered daily i.p. for four consecutive days, at day 5 after tumor cell implantation. TMZ was administered in a solution of 10% DMSO in 0.9% saline. Vehicle solutions were given to control groups.

#### Animal observation

Animals were monitored each day for weight loss, ataxia, and periorbital hemorrhage [48]. For study of EB1-overexpression consequences in glioma progression, three animals per group were sacrificed at post tumor transplant day 14, 21, and 28, and brains were removed and stored at −80°C.

#### Tumor volume evaluation

Frozen brains were serially sectioned using a Leica cryostat, and 20 μm sections were stained with Hematoxylin/Eosin (HE) for histomorphology and measures of the tumor volume. Images of HE-stained sections were captured with a Leica Z16APO macroscope using the Leica Application Suite 2.8.1 Software. The tumor area was manually outlined and measured using Image J software. Knowing the thickness and the number of sections, we calculated the total volume of each tumor. Tumor volumes were measured for three animals per group.

#### Assessment of tumor cell proliferation

Ki-67 immunostaining were performed on sections from paraffin embedded samples, with monoclonal Anti-Ki-67 antibody (clone MIB-1; Dako) and hematoxylin counter-staining. Three random representative fields within tumor areas were used for quantitation. Each Ki-67 positive cell was counted and normalized to the total number of cells in each field.

### Analysis of microtubule dynamics from EB3-GFP and CLIP 170-GFP comet data

U87-MG cells (6 × 10^4^ cells/well) were grown for 24 hours on 8-well Labtek II chamber slides (Labtek, Thermo Scientific, Roskilde, Denmark) precoated 1 hour with fibronectin (10 μg/ml). U87-MG cells were then transfected with plasmid coding for green fluorescent protein EB3-GFP or CLIP 170 GFP using lipofectamineTM 2000 system (Invitrogen). Before microscopy analysis, cells were incubated with various concentrations of VCR for 4 hours.

Time-lapse acquisitions for microtubule dynamic instability experiments were performed with a Leica DM-IRBE equipped with a 100X objective lens. Sixty images per cell were acquired at 2-s intervals using a digital camera (CCD camera Coolsnap FX; Princeton Instruments). EB3-GFP comets were detected using the plusTipTracker package [49]. The software package can be downloaded from http://lccb.hms.harvard.edu. Comet detection requires no user intervention, as the detection algorithm automatically estimates locally optimal thresholds. Tracking and inference of complete MT trajectories by plusTipTracker requires user-defined settings of several control parameters (Watershed-based detection: sigma 1 = 3; sigma 2 = 4 and *K* = 1; Tracking search radius range = 5–10 pixels, minimum subtrack length = 4 frames, maximum gap length = 12 frames, max shrinkage factor = 1.5, maximun angle forward = 30° and backward = 10°; fluctuation radius = 1.5 pixel ; Post processing frame rate = 2s; pixel size = 130 nm). Correct tracking was verified by visual inspection of several overlay movies. The parameter settings were kept identical for all movies. The plusTipTracker software package contains modules for the parameterization of trajectories characterizing MT dynamics within one movie and for the comparison of the dynamics between movies. Many of the metrics are redundant and thus cannot be considered for comparative analyses without further dimensionality reduction. To assess the consistency we chose 5 metrics for pair-wise statistical testing: mean growth speed, mean growth lifetime, mean growth length, time–based catastrophe frequency and distance-based catastrophe frequency. A t-test was performed for each of these metrics. For each experimental condition, 5 to 8 cells were analysed (400-1200 tracks per cell).

### Statistical analysis

Data are presented as mean ± SEM. Cell counting, cellular viability data, tumor volumes were analyzed by Student's t test. Overall survival (OS) was defined to be the time from GBM diagnosis to death from any cause, censored at the date of last contact. Progression-Free Survival (PFS) was defined to be the time from GBM diagnosis to progression or death, censored at the date of last contact. PFS and OS were estimated by the Kaplan-Meier method. The log-rank test was used to estimate survival distributions. Prognostic factors with *p* < 0.15 in univariate analysis were explored in multivariate analysis. Cox proportional hazards models were used for multivariate analyses and to estimate hazard ratios in regression models. Reported p-values are two-sided, and *p* < 0.05 was considered statistically significant. Asterisks indicate significant level vs control *p < 0.05; **p < 0.005; ***p < 0.001. Statistical analyses were performed with spss.version17® and GraphPad 5.0 statistical software.

## SUPPLEMENTARY FIGURES, TABLE, MOVIES


