# Supplementary material for: End-binding 1 protein overexpression correlates with glioblastoma progression and sensitizes to Vinca-alkaloids in vitro and in vivo
Source: Oncotarget. 2014 Nov 28;5(24):12769–87. doi: 10.18632/oncotarget.2646 (PMC4350359; doi:10.18632/oncotarget.2646)
Supplement: Supplementary file 1 [file oncotarget-05-12769-s001.pdf]

SUPPLEMENTARY FIGURES, TABLE, MOVIES

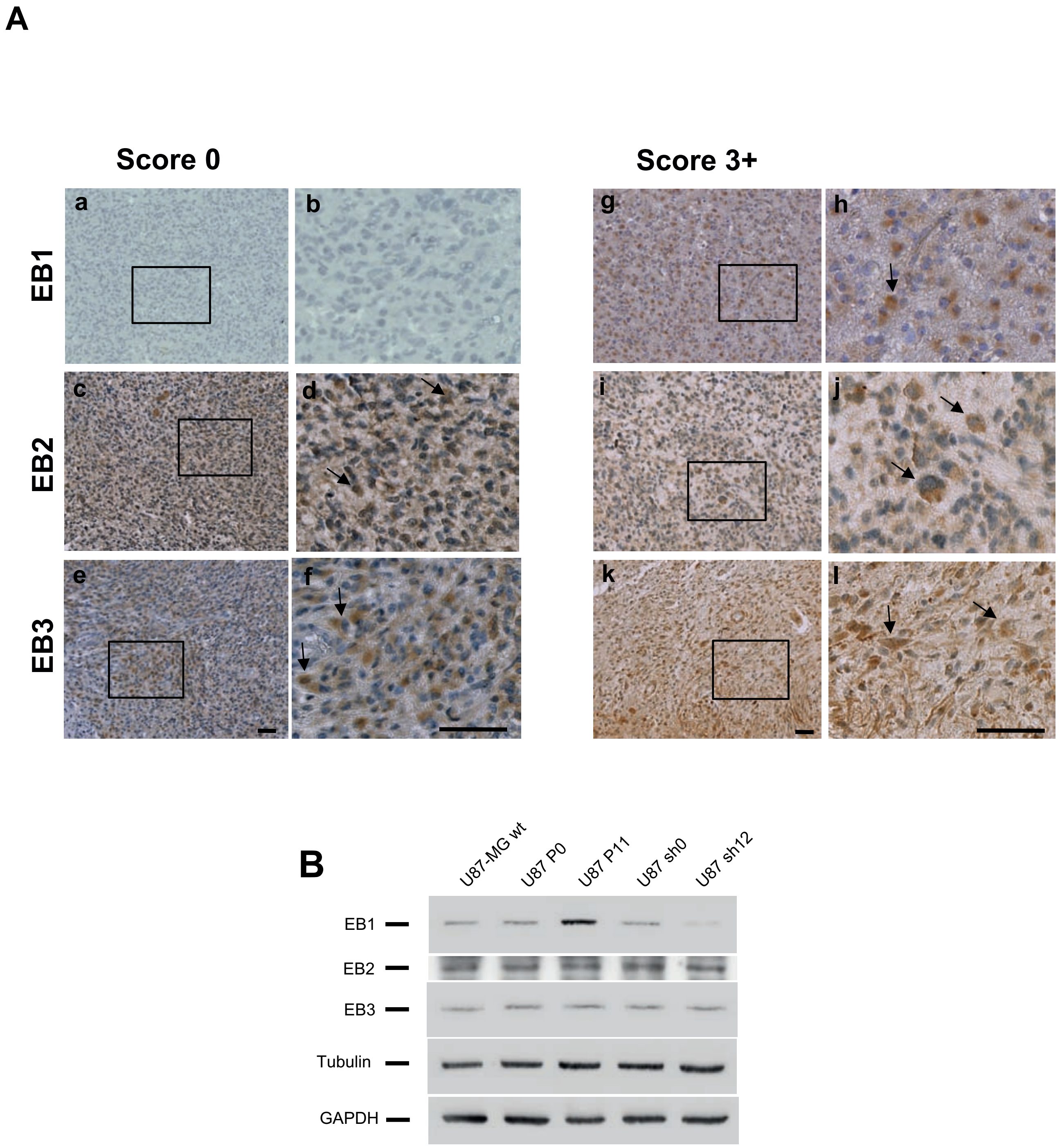

Supplementary Figure S1: Expression of EB2 or EB3 proteins according to EB1 scoring in glioblastoma. **(A)** Immunohistochemical analysis of expression of EB2 (c, d and i, j) and EB3 (e,f and k,l) in paraffin sections of glioblastoma tissue with low (score 0) or high expression (score 3+) of EB1 (a,b and g,h). Arrows on magnified images show the cytoplasmic localization of EB proteins in glioma cells (right columns). *Bar=50 µm*. **(B)** Western blot analysis of EB2 and EB3 levels in EB1 down-regulating clone (U87 sh12), EB1 overexpressing clone (U87 P11) or control clones (U87-MG wt, sh0 and P0).

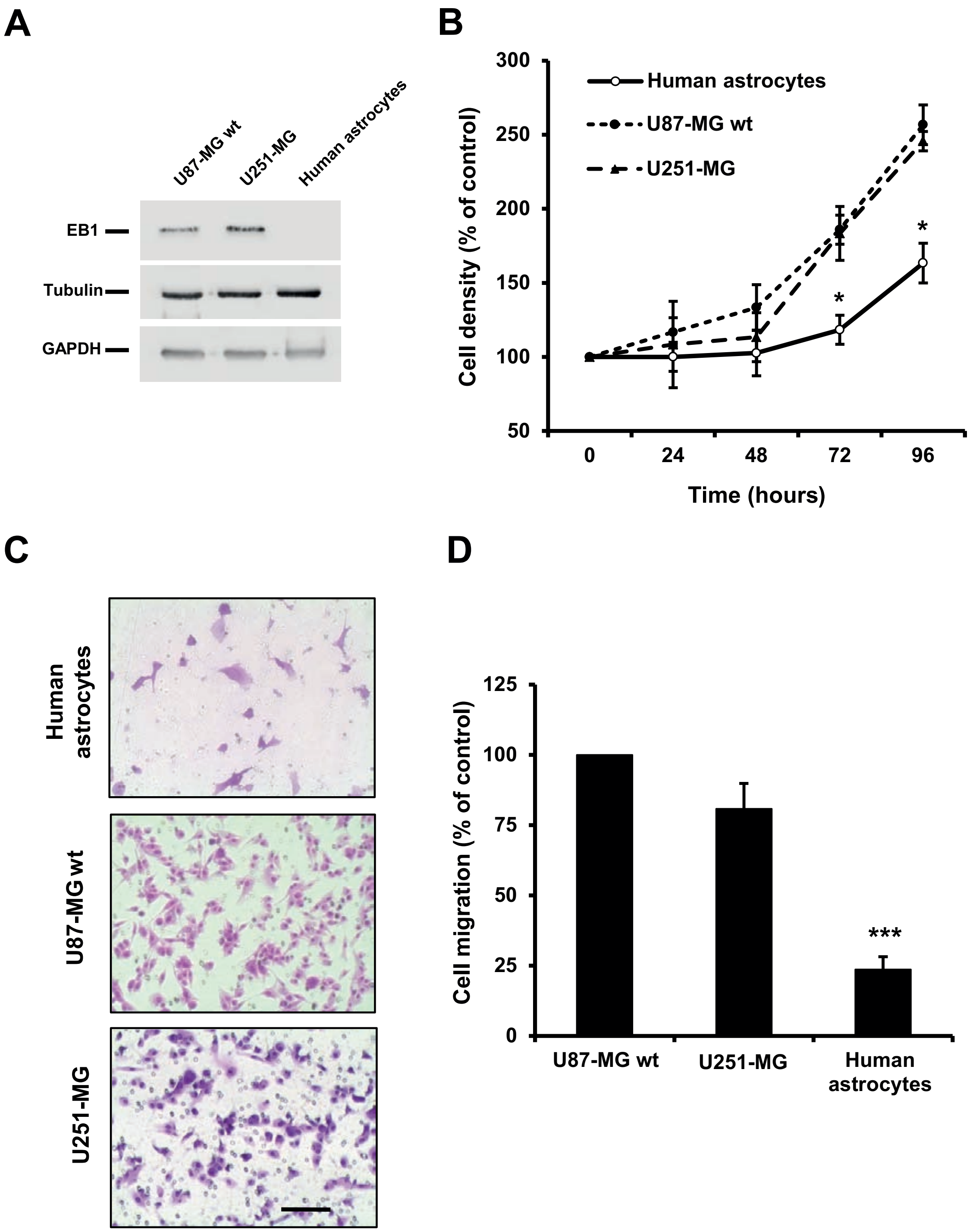

Supplementary Figure S2: undetectable level of EB1 expression in astrocytes is associated with poor migration and growth. (A) Analysis of EB1 expression level by Western blot of normal human astrocytes in comparison with U87-MG wt and U251-MG wt cells. **(B)** Sulforhodamine B cell density assay of normal human astrocytes in comparison with U87-MG wt and U251-MG wt cells. At each time, analysis were performed in sixplicates. At least three independent experiments were performed for each condition. Bar ± SEM. (*) indicates significant difference from U87-MG wt control cells:*$p < 0.05$. **(C)** Representative images of migratory normal human astrocytes in comparison with U87-MG and U251-MG cells, using the transwell migration assay (crystal violet staining, magnification of 100×). **(D)** Quantification of migratory cells, as determined by counting the cell number under the microscope with a magnification of 100×, in the transwell migration assay. At least three independent experiments were performed for each condition. Bar ± SEM. (*) indicates significant differences from U87 control cells: ***$p < 0.001$.

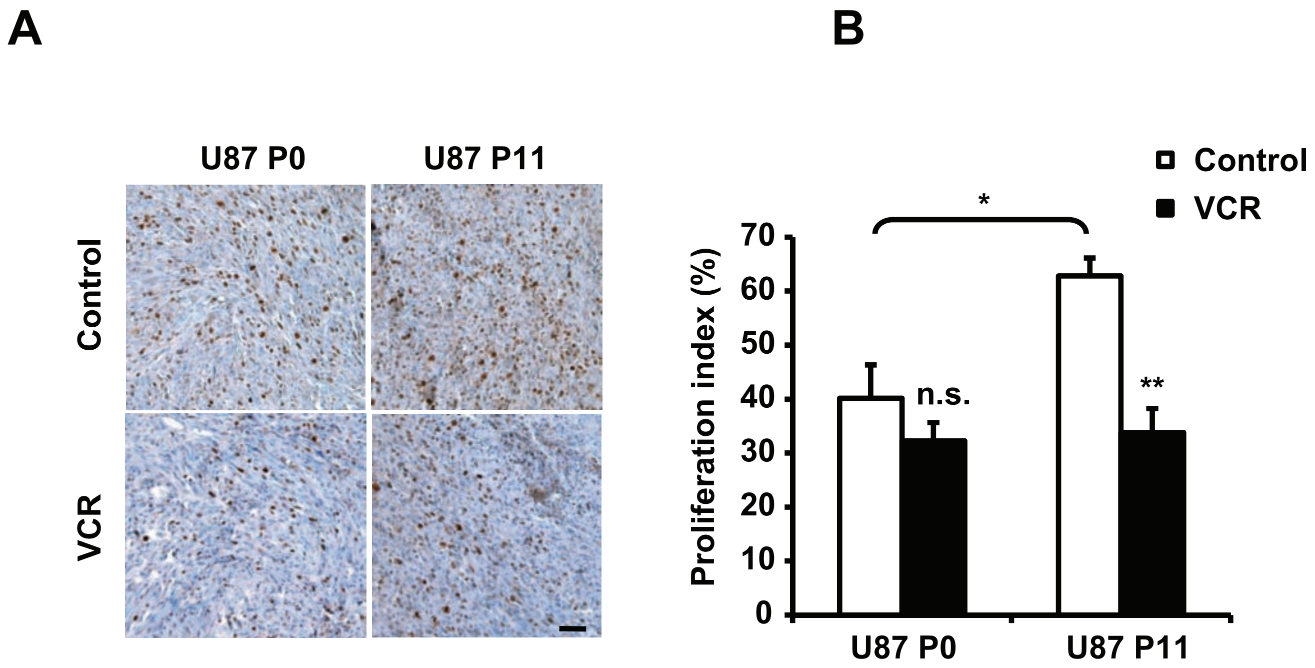

Supplementary Figure S3: EB1 overexpression increases proliferation rate of U87-MG cells *in vivo* and sensitizes glioblastoma tumors to VCR anti-proliferative effects. (A) Ki-67 staining of proliferative cells in EB1-overexpressing U87 P11 or control U87 P0 tumors and intravenously treated with VCR or vehicle. *Bar = 50 µm*. **(B)** Proliferation index in the four groups. Bar ± SEM. (*) indicates significant differences from control:*$p < 0.05$; ** $p < 0.005$; n.s.: non significant.

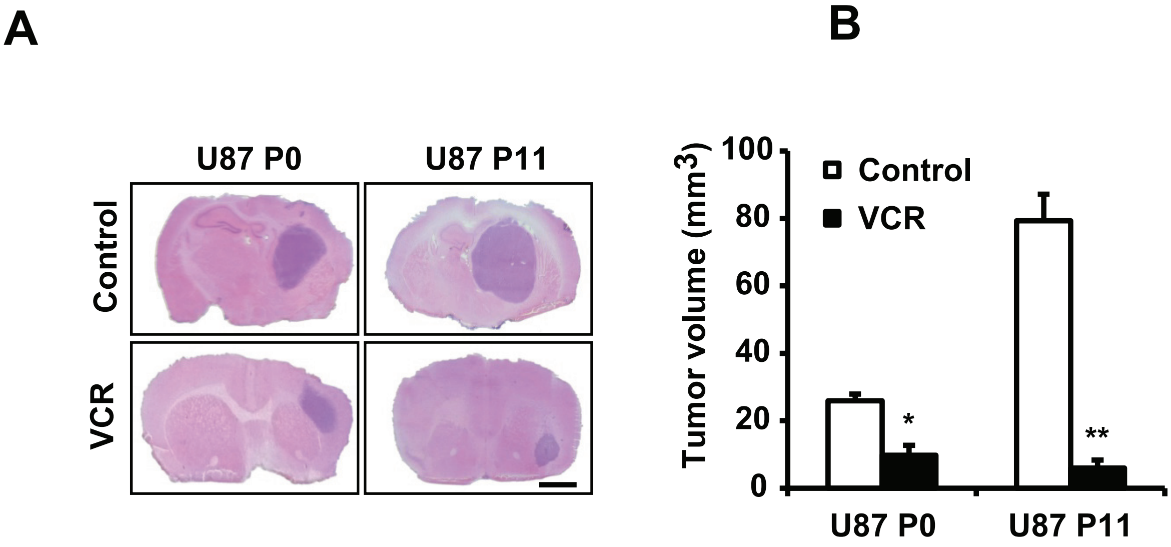

Supplementary Figure S4: EB1 overexpression sensitizes glioblastoma cells to *Vinca*-alkaloid anti-tumor growth effect. **(A)** Typical coronal sections of brains from mice intracerebrally grafted with EB1-overexpressing U87 P11 cells or control U87 P0 cells and intravenously treated with VCR or vehicle. Brains were recovered 21 days following cell transplantation. *Bar = 1 mm*. **(B)** Tumor volumes (mean ± SEM of 3 animals per treatment group). $^{*}p < 0.05$; $^{**}p < 0.005$.

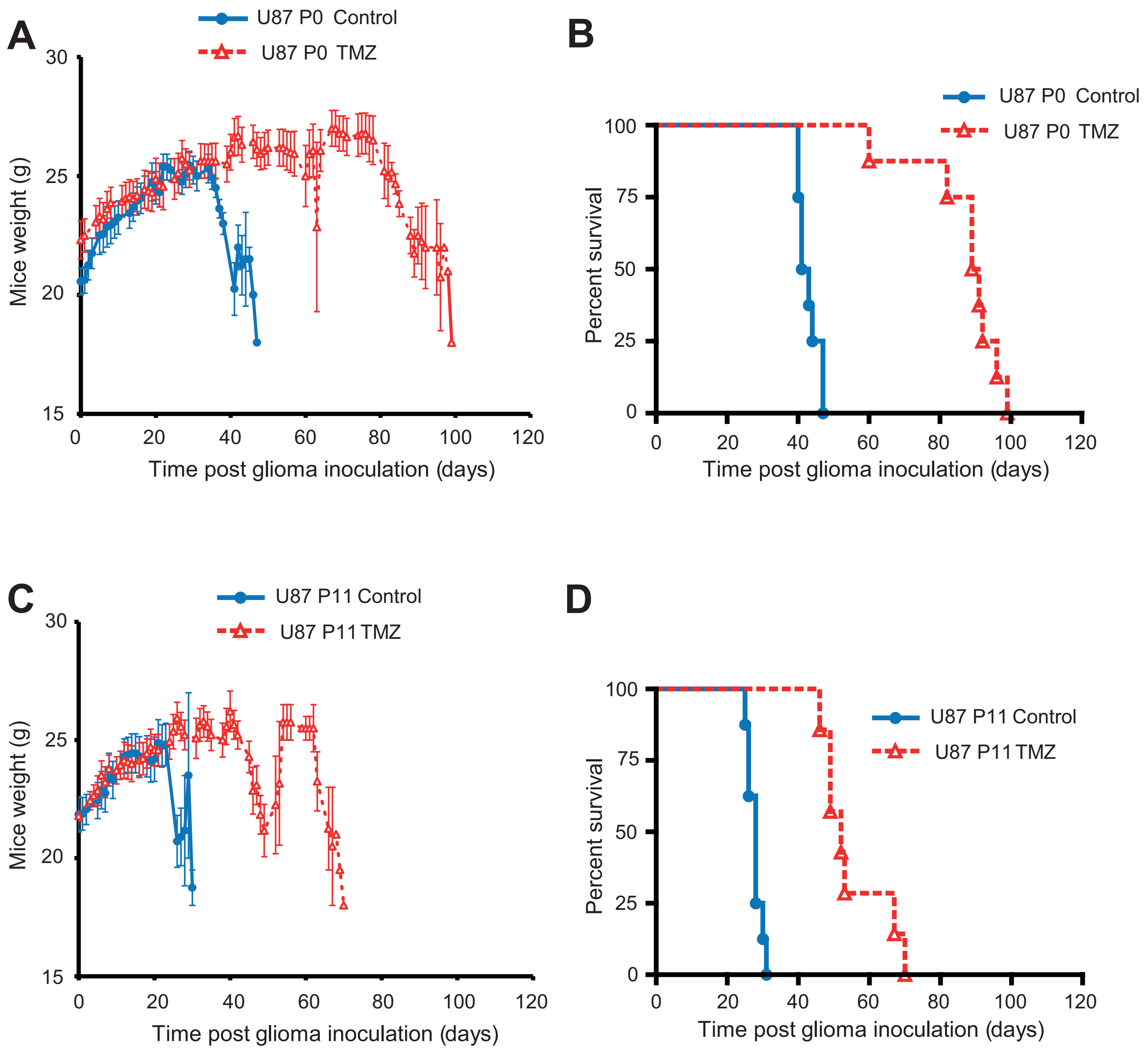

Supplementary Figure S5: EB1 overexpression does not sensitize glioblastoma cells to TMZ anti-tumor growth effect. Mean weights **(A, C)** and Kaplan–Meier survival plot **(B, D)** of control U87 P0 or EB1-overexpressing U87 P11 bearing mice intravenously treated with TMZ or vehicle. Bar ± SEM.

Supplementary Table S1. Estimate of Median OS and PFS according to EB1 glioblastoma sample scoring

EB1 score	Median OS			Median PFS		
	Estimate (months)	95% Confidence Interval		Estimate (months)	95% Confidence Interval	
		Lower Bound	Upper Bound		Lower Bound	Upper Bound
0	25.62	5.714	45.526	13.653	5.143	22.162
1	13.421	12.524	14.319	7.901	6.487	9.315
2	10.909	8.992	12.826	5.157	3.761	6.553
3	10.248	6.914	13.582	3.24	1.062	5.418

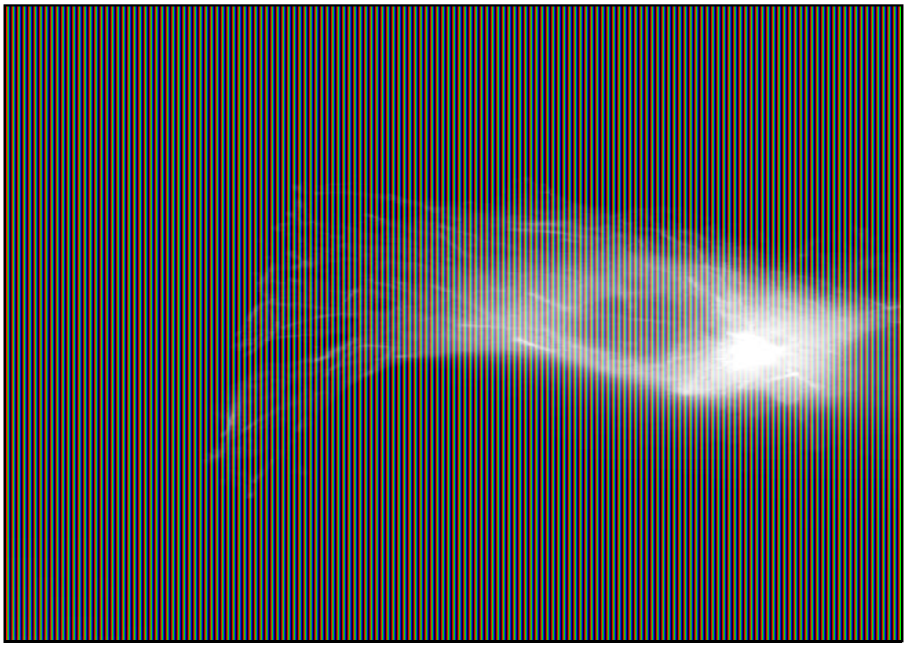

Supplementary Movie S1: U87-P0 clone EB3-GFP transfected, control (10 frames/s).

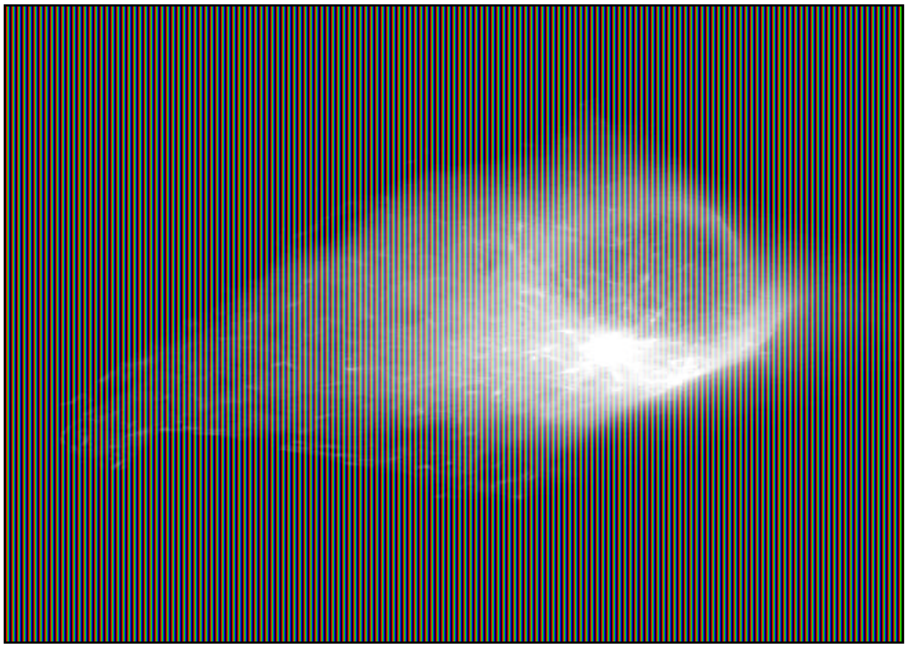

Supplementary Movie S2: U87-P0 clone EB3-GFP transfected, treated with 1.6 nM VCR for 4 h (10 frames/s).

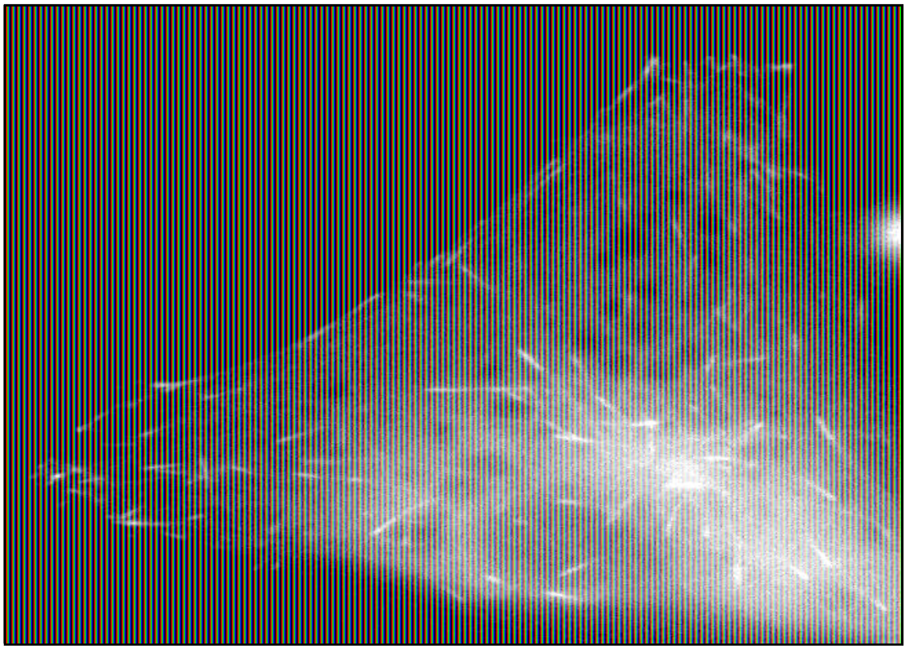

Supplementary Movie S3: U87-P11 clone overexpressing EB1, EB3-GFP transfected, control (10 frames/s).

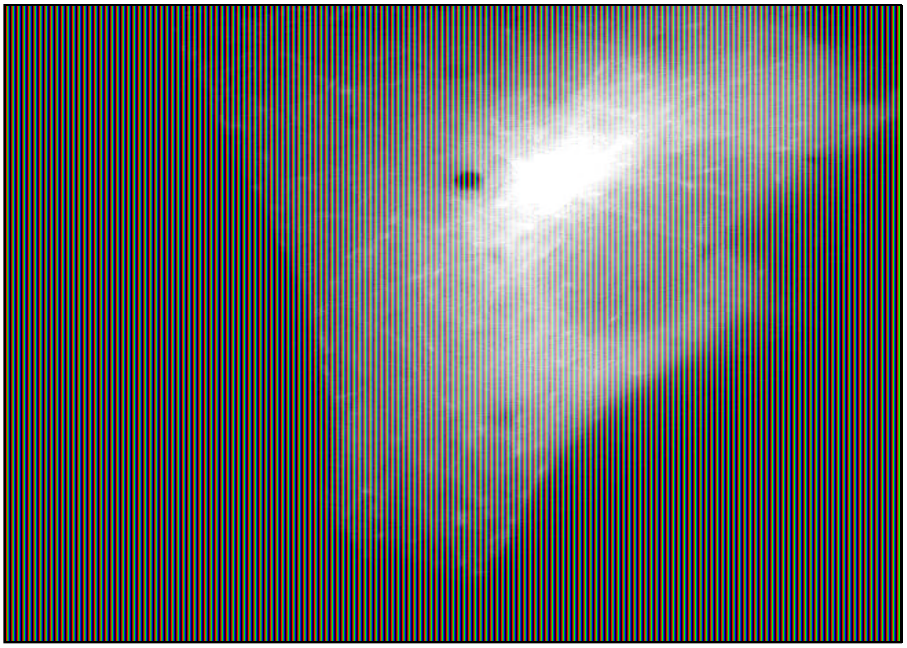

Supplementary Movie S4: U87-P11 clone overexpressing EB1, EB3-GFP transfected, treated with 1.6 nM VCR for 4 h (10 frames/s).
